# One-Carbon Metabolism: Biological Players in Epithelial Ovarian Cancer

**DOI:** 10.3390/ijms19072092

**Published:** 2018-07-19

**Authors:** Andrea Rizzo, Alessandra Napoli, Francesca Roggiani, Antonella Tomassetti, Marina Bagnoli, Delia Mezzanzanica

**Affiliations:** Unit of Molecular Therapies, Department of Research, Fondazione IRCCS Istituto Nazionale dei Tumori, Via Amadeo 42, 20133 Milan, Italy; andrea.rizzo@istitutotumori.mi.it (A.R.); alessandra.napoli@istitutotumori.mi.it (A.N.); francesca.roggiani@istitutotumori.mi.it (F.R.); antonella.tomassetti@istitutotumori.mi.it (A.T.); marina.bagnoli@istitutotumori.mi.it (M.B.)

**Keywords:** one-carbon metabolism, epithelial ovarian cancer, choline metabolism, folate, folate receptor alpha

## Abstract

Metabolism is deeply involved in cell behavior and homeostasis maintenance, with metabolites acting as molecular intermediates to modulate cellular functions. In particular, one-carbon metabolism is a key biochemical pathway necessary to provide carbon units required for critical processes, including nucleotide biosynthesis, epigenetic methylation, and cell redox-status regulation. It is, therefore, not surprising that alterations in this pathway may acquire fundamental importance in cancer onset and progression. Two of the major actors in one-carbon metabolism, folate and choline, play a key role in the pathobiology of epithelial ovarian cancer (EOC), the deadliest gynecological malignancy. EOC is characterized by a cholinic phenotype sustained via increased activity of choline kinase alpha, and via membrane overexpression of the alpha isoform of the folate receptor (FRα), both of which are known to contribute to generating regulatory signals that support EOC cell aggressiveness and proliferation. Here, we describe in detail the main biological processes associated with one-carbon metabolism, and the current knowledge about its role in EOC. Moreover, since the cholinic phenotype and FRα overexpression are unique properties of tumor cells, but not of normal cells, they can be considered attractive targets for the development of therapeutic approaches.

## 1. Introduction

Cancer is a disease of uncontrolled growth, in which cells undergo genetic and epigenetic changes that affect critical cellular functions. Accumulating evidence suggests that oncogenic signaling is linked to multiple metabolic rearrangements that confer selective advantages to cancer cells, allowing them to proliferate outside the context of normal tissue development and to meet the requirements for sustaining a transformed cellular steady state. The relationship between metabolism and cancer is well known, and a state of deregulated cellular metabolism is an established hallmark of cancer [1,2]. Over the past several years, a comprehensive description of metabolic modifications emerged from studies on different tumors, highlighting profound alterations in pathways that improve tumor survival and malignant growth. Besides the clear involvement of glucose and glycolysis in sustaining cancer energy metabolism (the Warburg effect) [3,4], amino-acid metabolism involving serine, glycine, and the one-carbon units they provide increases the number of metabolic pathways essential for cancer progression [5]. Moreover, metabolic reprogramming and metabolites themselves can interfere with oncogenic-driven cell signaling and cancer transformation. These alterations can be evaluated as likely therapeutic targets to develop novel approaches to improve the efficacy of standard chemotherapeutic treatments [6,7]. In this review we highlight the metabolism and metabolites involved in the one-carbon cycle in epithelial ovarian cancer (EOC), also identify possible therapeutic applications.

## 2. EOC Biology and Metabolic Alterations

### 2.1. Pathobiology

EOC is the main cause of death from gynecological cancer, and this is mainly attributable to late diagnosis and the development of chemoresistance [8]. Most EOC patients present with advanced-stage disease due to the lack of specific symptoms, adequate diagnostic tools, and screening strategies [9]. Standard treatment for advanced-stage EOC is tumor-debulking surgery, followed by adjuvant platinum-based chemotherapy [10]. Despite a good response rate to front-line treatment, about 70% of patients relapse within two years, and the majority eventually develop an incurable state of platinum-resistant disease with a five-year survival rate that is still below 40% [10]. EOC is a highly heterogeneous disease that, from a pathological and molecular point of view, can be classified as a type I or type II tumor [11]. Type I EOCs are typically well differentiated, characterized by overall genomic stability and the absence of *TP53* mutations. Type II tumors are more aggressive, characterized by *TP53* mutations, high genomic instability, frequent mutations in *BRCA1* or *BRCA2*, and deficiencies of the homologous recombination pathway [12]. However, excluding *BRCA1*/*2* mutations guiding the use of PARP inhibitors [13,14], all EOCs are still treated with the same therapeutic modalities, and the identification of molecular prognostic and predictive biomarkers is still an unmet clinical need.

### 2.2. Metabolic Alterations

Like many other cancer types, EOC shows alterations in the main metabolic pathways, including glycolysis, the tricarboxylic acid cycle, as well as lipid and amino-acid metabolism. These metabolic pathways are interconnected, and their deregulation contributes to cancer onset and progression [15]. Other modifiable metabolic abnormalities such as obesity, type II diabetes mellitus, and metabolic syndrome were recently associated with EOC incidence and poor outcomes [16,17,18,19]. The mechanisms via which these metabolic derangements contribute to increased cancer risk and mortality are multifactorial and not completely understood. An excess of adipose tissue was associated with dysregulation of adipokine and cytokine levels [20]. Such altered chemokine expression patterns may affect immune responses, and ultimately, favor tumor immune evasion [21].

Alterations in lipid metabolism can be considered a key feature involved in the interaction with the tumor microenvironment, and, as in other malignancies, increased lipid synthesis is important for the pathogenesis of EOC [22]. Through adipokine secretion, adipocytes present in the peritoneal omentum contribute to the metastatic cascade by homing EOC cells to the omentum, where they give rise to secondary localizations. Furthermore, adipocytes provide fatty acids to cancer cells, promoting rapid tumor growth [23]. Recently, the observation of enhanced lipid metabolism and adaptation to starvation of EOC cells grown in suspension compared to adherent cells suggested that floating tumor cells present in EOC patients’ ascites need this specific metabolism in order to grow [24]. Alteration of lipid metabolism was also detected in EOC patients at both early and late disease stages, as well as in patients with recurrent disease [25]. Fatty-acid synthase expression was found to be upregulated in EOC, and this was correlated with a poor prognosis [26,27,28].

Many tumor types, including EOC, were shown to reprogram their metabolism during progression, switching from oxidative phosphorylation to glycolysis, a phenomenon known as the Warburg effect [3,4]. This metabolic phenotype is frequently associated with alterations of the p53 and PI3K/Akt/mTOR pathways that are commonly detected in EOCs, and that are known to be associated with chemoresistance [29]. Increases in glucose and glutamine metabolism following platinum treatment were observed in EOC models, suggesting that tumor cells under the pressure of drug treatment may further reprogram their metabolism to improve their fitness and survival capability [30]. Recent studies showed that EOC cells, according to their in vitro viability under glucose starvation, can be categorized into glucose-deprivation sensitive (glucose addicted, GA) and glucose-deprivation resistant (glucose non-addicted, GNA). Interestingly, EOC patients with a GA phenotype have significantly better progression-free survival (PFS) than GNA patients [31]. Aberrant glutamine metabolism, with overexpression of glutaminase, was associated with poor survival in EOC patients and platinum resistance in EOC cellular models [32]. Interestingly, analysis of patient-derived EOC cell lines revealed the same correlation between chemoresistance and a highly metabolic phenotype [33].

The importance of metabolic alterations in cancer cell survival promoted studies to exploit such alterations as new diagnostic and prognostic biomarkers for EOC. Metabolomic analysis of the sera of EOC patients and controls was suggested as a possible diagnostic tool for early-stage EOC [34], and a new diagnostic marker, 27-nor-cholestane pentol, complementary to CA125, was identified using a two-step metabolomic approach [35]. This technology was applied to discriminate EOC patients from healthy donors, as it identified a disturbed histidine–nucleotide superpathway in plasma samples from EOC patients [36]. A number of metabolomic analyses were performed on body fluids from healthy donors, patients with borderline tumors, and patients with EOC, yielding promising results for patient classification [37,38]. Metabolic analysis revealed 53 metabolites to be specific biomarkers for EOC, including piperine, 3-indolepropionic acid, 5-hydroxyindoleacetaldehyde, and hydroxyphenyllactate [39]. Alterations of several metabolic pathways, such as histidine and tryptophan metabolism, arginine biosynthesis, arginine and proline metabolism, and alanine, aspartate, and glutamine metabolism, were found in the sera of EOC patients [40]. Moreover, metabolic analysis of 98 plasma samples defined kynurenine, acetylcarnitine, phosphatidylcholine, and lysophosphatidylethanolamine as potential predictive biomarkers to distinguish short-term from long-term EOC survivors [41].

## 3. One-Carbon Metabolism in Physiology and Cancer

**Figure 1:**
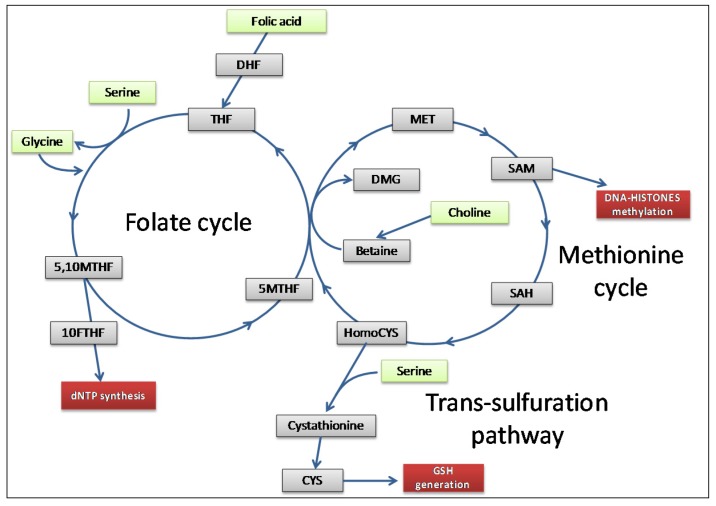
Simplified overview of the main fluxes involved in one-carbon metabolism (inspired by reference [44]). One-carbon cycles act as a biochemical network to sustain fundamental physiological functions, overall DNA and histone methylation, dNTP synthesis, and GSH generation (red rectangles). Several molecular inputs (green rectangles) actively fuel the pathway, generating a number of intermediate metabolites (grey rectangles) through which carbon units are cycled. Abbreviations: dihydrofolate (DHF); tetrahydrofolate (THF); methyltetrahydrofolate (MTHF); formyltetrahydrofolate (FTHF); dimethylglycine (DMG); methionine (MET); S-adenosylmethionine (SAM); S-adenosylhomocysteine (SAH); cysteine (CYS); glutathione (GSH).

One-carbon metabolism comprises complex biological networks in which input nutrients are processed through a series of chemical reactions to cycle carbon units. The produced metabolites are then made available for important processes including cellular biosynthesis, methylation, regulation of redox status and amino-acid homeostasis [42]. Essentially, one-carbon metabolism involves three pathways: the folate and methionine cycles, and the trans-sulfuration pathway [43] (Figure 1).

Quantitatively, folic acid, serine, glycine and choline are the most important metabolites through which cells refuel the one-carbon metabolism [44]. Folic acid is the driver molecule of the folate cycle that enters the network after its reduction to tetrahydrofolate (THF). Serine is the main cellular donor of one-carbon units that through its conversion into glycine allows THF to be converted in methyltetrahydrofolate (MTHF) [45]. Glycine itself is a potential source of one-carbon units that can be derived via a process called the glycine cleavage system [46]; it can also function as a precursor for glutathione (GSH) and serine synthesis. Besides folic acid, other B-vitamins (B2, B6, and B12) are required to steadily maintain the one-carbon flux, playing a fundamental role in this context by acting as coenzymes and methyl-group acceptors [47].

The folate and methionine cycles overlap upon the synthesis of MTHF necessary for the generation of methionine through methylation of homocysteine [48]. Methionine is then converted into the fundamental metabolite S-adenosylmethionine (SAM), the universal cellular methyl donor required for DNA, RNA, protein, and lipid methylation. For this reason, SAM represents the second most common enzymatic cofactor after ATP in cell biochemistry [49]. The trans-sulfuration pathway is, hence, connected to the methionine cycle through homocysteine [48].

Another important input metabolite of one-carbon metabolism is choline. Choline is a quaternary amine involved in key biochemical pathways, considered an essential nutrient that cells use as a building block for a variety of metabolites including betaine, acetylcholine, and phosphatidylcholine (PtdCho) [50]. While free choline is primarily used in phospholipid synthesis, choline excess is the source of one-carbon units for methylation. Upon oxidation to betaine, choline can directly re-methylate homocysteine (independent of THF), thus contributing to methionine formation and homeostasis [44].

Although one-carbon metabolism was long considered a housekeeping process given its physiological function of regulating the flow of carbon units, several indications closely link it to cell transformation [51]. Due to its importance in satisfying biosynthetic demands, tumor cells can therefore alter or become more dependent on one-carbon metabolism to sustain their own proliferation [51]. Any oncogenic impact on this pathway can be pervasive, and it may induce a cascade of alterations in other downstream networks. Reductions or increases in metabolic flows cause changes in the biochemical reactions involved in cell homeostasis, as well as variations in metabolite levels, thus influencing pathways in which each single metabolite is involved [52]. Metabolic enzymes that regulate the rate-limiting steps of these reactions are often responsible for cancer-associated metabolic aberrations [15]. To date, over 5000 sequence variations in one-carbon pathway genes, including folylpolyglutamate synthase (*FPGS*), methylenetetrahydrofolate reductase (*MTHFR*), methionine synthase (*MTR*), methionine synthase reductase (*MTRR*), and serine hydroxymethyltransferase 2 (*SHMT2*), were described [53]. All these observations suggest that one-carbon metabolism deregulations are possible drivers of oncogenesis and cancer progression [5,44,54,55,56]. Essentially, one-carbon metabolism generates three main output processes important for cancerogenesis and tumor cell survival: DNA and histone methylation, purine/pyrimidine synthesis, and the maintenance of redox status [43] (Figure 1).

### 3.1. DNA and Histone Methylation

In the epigenetic context, one-carbon metabolism provides methyl groups for methylation reactions involving both DNA and histone proteins [57].

Histone methylation, which significantly contributes to chromatin architecture and gene-expression dynamics, is the best characterized post-translational modification. Methylation can either induce or repress gene transcription, and it can occur on residues of histidine, arginine, or lysine of the H3 and H4 histones via the transfer of methyl groups from SAM [58].

DNA methylation is one of the main epigenetic features recognized as a repressive mark associated with a decrease in DNA accessibility, causing the absence of transcription [59]. This process, catalyzed by several methyltransferase enzymes, allows the post-replicative attachment of a methyl group from SAM at the 5′ position of cytosines within a context where these are followed by guanines (CpG dinucleotides). DNA methylation, being a crucial (epi)regulator of gene expression, is a dynamic process with fundamental relevance for cellular behavior [60,61]. In cancer, two opposite epigenetic phenomena related to DNA methylation may occur: (1) aberrant expression of elements like oncogenes, as a result of demethylation [62]; (2) silencing of genes that regulate critical cellular functions, as a result of de novo methylation in CpG islands [63,64,65]. In EOC, CpG-island methylation contributes to the down-modulation of important genes regulating the cell cycle, apoptosis, and cell adhesion [66]. The DNA repair pathway, of which *BRCA1* is a component, can also be affected by CpG-island methylation [67]. Since the evolution of genome-wide technical approaches, more recent studies showed a pattern of genomic CpG methylation among the various histological types and grades of EOC [68,69], and also between chemoresistant and chemosensitive tumors [70].

### 3.2. Trans-Sulfuration and GSH

The trans-sulfuration pathway is a biochemical network linked to the methionine cycle through the intermediate homocysteine, and is specifically involved in the biosynthesis of cellular redox-controlling molecules [71]. The critical product of trans-sulfuration is GSH, a tripeptide with a strong reducing-nucleophile power, representing the major cellular antioxidant molecule [72]. In addition to participating in efflux transporter-mediated detoxification [73], GSH is a master regulator of oxidative-stress homeostasis, able to prevent cell damage due to reactive oxygen species [74]. It may negatively interfere with alkylating drugs and radiation treatments, which are specifically designed to damage DNA and promote cell death through the induction of high oxidative stress.

High levels of GSH could, therefore, be involved in chemoresistance in many types of cancer [75,76,77]. GSH is known to mediate resistance to both cisplatin and carboplatin (two drugs used in the front-line treatment of EOC) through several mechanisms, such as drug-uptake reduction and increased intracellular drug detoxification/inactivation, increased DNA repair, and inhibition of drug-induced oxidative stress [78]. Different studies in EOC showed higher levels of GSH in tumor tissue than in their normal counterparts, and a correlation between intracellular GSH content and chemoresistance [79,80,81].

### 3.3. DNA Biosynthesis

Nucleotides, required in every single cell for de novo DNA and RNA synthesis, are among the main molecular outputs of one-carbon metabolism. Different steps in the biosynthesis process of pyrimidine and purine nucleotides need several cofactors generated via one-carbon-metabolism-related pathways [82]. In particular, through a series of reversible reactions 5,10MTHF is converted into 10-formyl tetrahydrofolate, the methyl donor substrate for de novo purine (dAMP, dGMP) backbone formation. MTHF is also used directly to convert the pyrimidine dUMP into dTMP. Given the high proliferation rate of cancer cells, and therefore, the requirement of nucleotides, cancer cells have a high demand for one-carbon units [44].

## 4. One-Carbon-Metabolism-Dependent Processes and EOC

**Figure 2:**
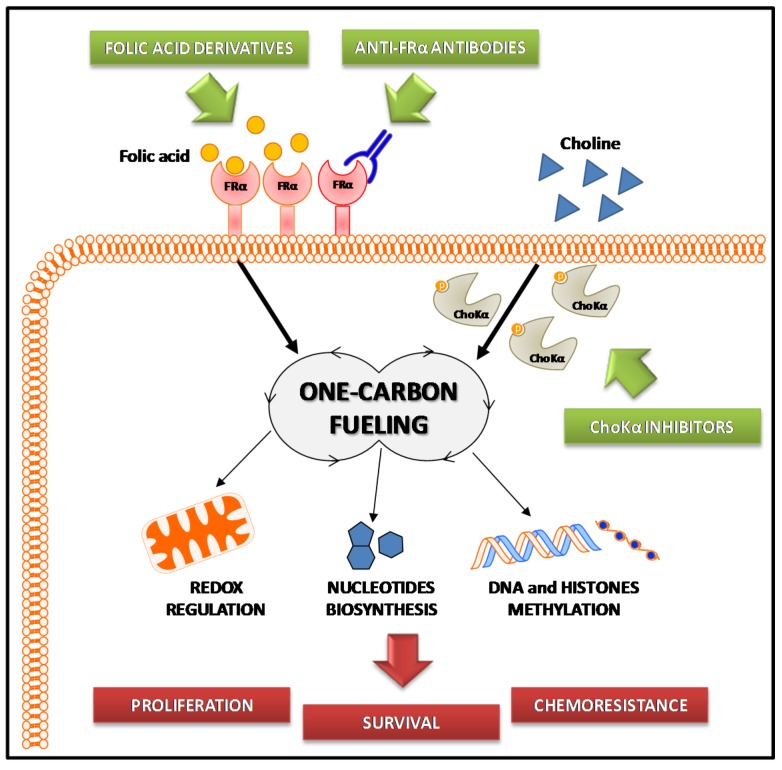
Scheme of the involvement of one-carbon metabolism in epithelial ovarian cancer (EOC). The folic acid and choline metabolisms are deregulated in EOC due to overexpression of the alpha isoform of the folate receptor (FRα) and hyperactivation of choline kinase alpha (ChoKα), respectively. Allowing cell proliferation, chemoresistance, and survival (red rectangles), these processes contribute to EOC transformation and aggressiveness. Strategies targeting alterations in folate and choline metabolism (green rectangles) may, thus, be promising alternative approaches to treat EOC.

Two of the major one-carbon metabolism players, folate and choline (see Figure 1), have a significant role in EOC biology. Indeed, EOC is characterized by a cholinic phenotype [83] and by overexpression of the high-affinity alpha isoform of the folate receptor (FRα) [84]. These two aspects, summarized in Figure 2, are analyzed in the paragraphs below.

### 4.1. Choline Metabolism

Clinical and molecular approaches, such as magnetic resonance spectroscopy and nuclear magnetic resonance, strongly indicate an alteration of choline metabolism in several cancer types including EOC [85]. An increased content of choline-containing metabolites, phosphocholine (PCho) in particular, is sustained by the overexpression and hyperactivation of choline kinase alpha (Chokα/*CHKA*) [86,87,88,89,90,91,92,93]. Chokα is a phosphotransferase that catalyzes choline phosphorylation to form PCho, the first reaction of the Kennedy Pathway (PtdCho biosynthetic pathway) [94]. ChoK is represented by three isoforms in mammalian cells, encoded by two separate genes: *CHKA* and choline kinase beta (*CHKB*). However, only Chokα has a central role in sustaining the PtdCho biosynthesis required for cell growth, while Chokβ alone cannot compensate for this activity [94]. Notably, a large body of evidence highlighted a fundamental contribution of Chokα to sustaining the transformed phenotype of EOC cells [90,95,96] beyond its physiological role in providing PCho for the synthesis of membrane lipids.

Different approaches in *CHKA* silencing, i.e., transient and stable RNA interference in EOC cell lines, showed a decrease in proliferation and an impaired capability of tumor cells to migrate and invade [96]. These observations clearly locate *CHKA* in metabolic-related networks that sustain EOC aggressiveness. As already mentioned, given that choline metabolism is closely connected to the one-carbon cycle, it can be hypothesized that the cholinic phenotype may play a fundamental role in EOC biology through the aberration of one-carbon-related functions. In line with this, metabolomic analysis upon *CHKA* knockdown in EOC in vitro models showed differences in the intracellular content of several metabolites related to one-carbon cycles such as methionine, cysteine, and GSH, which showed the most conspicuous alterations [97]. Moreover, a reduction in GSH content in *CHKA*-silenced cells was found to increase the sensitivity of EOC cells to standard chemotherapeutic agents such as platinum and doxorubicin, likely due to a loss of redox homeostasis [97], thus linking a sustained choline metabolism to EOC chemoresistance.

Of note, interference with *CHKA* expression was proven not to be deleterious for primary normal or immortalized non-tumorigenic surface-epithelial ovarian cells, suggesting that different responses are triggered in transformed versus non-transformed systems [97]. The cholinic phenotype, by recapitulating EOC addiction to GSH content for the maintenance of antioxidant defense, can be considered, therefore, a unique feature of cancer cells, and a suitable target to improve chemotherapeutic efficacy. This observation is relevant for possible therapeutic approaches. Impairing choline metabolism by targeting *CHKA* could be an attractive strategy which selectively affects the growth and survival of EOC cells while sparing normal epithelia. Synergism of *CHKA* knockdown with conventional treatment might open an interesting clinical perspective, since it could represent an alternative strategy to increase the treatment efficacy by also reducing the clinical dose of drugs and limiting the damage to normal cells [97].

Several inhibitors—mainly choline mimetics—were recently developed and tested in other solid tumors [98,99]; however, assessment of their efficacy in EOC still awaits the development of preclinical studies.

### 4.2. Folate Metabolism

Folic acid is a vitamin essential for normal proliferating cells. It is involved in epigenetic regulation and genome stability through the methylation processes, and it is necessary for the biosynthesis of purine and pyrimidine nucleotides that are essential for DNA and RNA synthesis [47]. Deregulated folate metabolism was associated with embryonic developmental disorders, brain defects, and cardiovascular diseases [100]. It is implicated in the development of cancer through alterations in DNA methylation and the disruption of DNA integrity and repair, thus interfering with the expression of critical tumor suppressor genes, such as *TP53*, and proto-oncogenes, such as *MET* [101,102].

Folate is a lipophilic molecule that is actively transported into the cell via three different systems: the reduced folate carrier (RFC) [103], the proton-coupled folate transporter [104], and the FR [105]. Three FR isoforms were identified, the α, β, and γ isoforms, each with a specific tissue distribution [106]. The most extensively studied isoform is FRα, which has a low expression level in normal cells, except those of the placenta and proximal renal tubules, but is highly expressed in various tumors of epithelial origin, such as mesothelioma [107], lung cancer [108], and EOC [109,110,111].

FRα may sustain tumor progression by increasing folate uptake or by generating regulatory signals supporting malignant cell proliferation and chemoresistance [112,113,114,115]. Recent studies showed that FRα overexpression in serous EOC samples was correlated with short PFS and poor prognosis [116]. Specifically, FRα appeared to modulate the response to chemotherapy through the induction of apoptosis resistance and the down-modulation of the proapoptotic molecules, BCL-2 and Bax [116]. Furthermore, indirect evidence demonstrated that folate is involved in other critical EOC processes, such as migration and invasion through increased FRα functionality, followed by reduction of E-cadherin expression [117].

As far as targeting of the folate metabolism is concerned, two general approaches can be adopted: a direct therapeutic approach to antagonize folate activity [118], or the exploitation of FRα as a target to redirect inhibitors to tumor cells [119] (Figure 2).

Since folate is necessary for DNA methylation, antifolate compounds inhibiting DNA methylation, such as methotrexate (MTX) and pemetrexed, are used as epigenetic regulators and cell proliferation inhibitors in anticancer therapy [120]. MTX is a potent inhibitor of dihydrofolate reductase, while pemetrexed is a multitarget antifolate inhibitor that showed efficacy in platinum-resistant EOC patients in a phase II clinical trial [121]. Miotti and colleagues reported that, in EOC cells, the internalization of physiological folate depends not only on the level of FRα expression, but also on the expression of the RFC [122]; the authors argued that this carrier may be a further transport vehicle of pemetrexed and folate analogs into malignant cells.

Due to its homogeneous and preferential distribution on tumor cells, FRα is an ideal tumor-associated antigen suitable as a therapeutic target [123]. Targeting of FRα with therapeutic molecules may be obtained via their binding to either folic acid or antibodies recognizing the receptor itself. Vintafolide is an example of an FRα-targeting agent obtained through conjugating folate to a derivative of the chemotherapeutic agent, vinblastine [124]; it was recently used in a phase II trial in combination with pegylated liposomal doxorubicin for the treatment of platinum-resistant EOC patients [125].

Many monoclonal antibodies directed against FRα are used as targeting agents [126]. Among the first to be isolated, MOv18 and MOv19 were generated via the immunization of mice with a surgical sample of human EOC [127]. The FRα-specific variable regions were then cloned in suitable vectors carrying the genes for the human immunoglobulin constant regions to generate their chimeric version [128]. MOv18 and MOv19 chemical or engineered derivatives are used in various diagnostic and therapeutic applications [129], and their potential is also exploited for use as immunotherapeutic agents [130,131,132,133,134]. Other anti-FRα antibodies that have reached clinical application are the humanized antibody, farletuzumab, which has entered a clinical trial in combination with carboplatin and taxane in patients with platinum-sensitive recurrent EOC [135], and mirvetuximab soravtansine (IMGN853), an antibody–drug conjugate affecting cell mitosis that showed positive tolerability when administered as monotherapy in EOC patients with platinum-resistant relapse [136].

## 5. Conclusions

The involvement of one-carbon metabolism in sustaining cancer cell survival is long recognized. However, its crosstalk with the epigenome, its involvement in genome maintenance, and its control of redox status and anabolic metabolism are just beginning to be understood.

It is only in recent years that a number of studies refocused their attention on this pathway, starting to provide new functional and mechanistic insights that could possibly be exploited to improve our ability to treat cancer. Indeed, antifolate compounds are being extensively used in therapy, although detrimental effects on normal cells and the development of resistance are common. Thanks to the newly acquired knowledge, future therapeutics are expected to more selectively target and inhibit specific functions, individual enzymes, and metabolites, to which cancer cells, but not normal cells, are addicted. Also, integrative metabolomic and bioinformatic approaches will possibly enable rapid progress in this area.

EOC is of particular interest in this setting because of its concurrent alteration in two pathways which are intimately involved in the one-carbon network, both providing carbon units as inputs: choline metabolism and folate internalization. In particular, the cholinic phenotype and increased FRα membrane expression might represent a complex dynamic strategy adopted by EOC cells to synergistically meet the one-carbon requirements to sustain and promote their growth and survival. Given the tumor’s addiction to choline metabolism and the tumor-restricted increased FRα expression, they both represent attractive targets for new therapeutic approaches based on their combination with known chemotherapeutic or targeted agents.

## Abbreviations

EOC	Epithelial ovarian cancer
FR	Folate receptor
GA	Glucose addicted
GNA	Glucose non-addicted
PFS	Progression-free survival
MTHF	Methyltetrahydrofolate
THF	Tetrahydrofolate
GSH	Glutathione
SAM	S-adenosylmethionine
PtdCho	Phosphatidylcholine
PCho	Phosphocholine
Chok	Choline kinase
RFC	Reduced folate carrier
MTX	Methotrexate
